# Benefits of physical activity on reproductive health functions among polycystic ovarian syndrome women: a systematic review

**DOI:** 10.1186/s12889-023-15730-8

**Published:** 2023-05-12

**Authors:** Muhammad Salman Butt, Javeria Saleem, Rubeena Zakar, Sobia Aiman, Muhammad Zeeshan Khan, Florian Fischer

**Affiliations:** 1 Department of Public Health, University of the Punjab, Lahore, Pakistan; 2 Akhtar Saeed Medical and Dental College, Lahore, Pakistan; 3 University of Child Health, Lahore, Pakistan; 4 Institute of Public Health, Charité – Universitätsmedizin Berlin, Berlin, Germany

**Keywords:** Exercise, Physical activity, Polycystic syndrome, Reproduction

## Abstract

**Background:**

Polycystic ovary syndrome (PCOS) is among the predominant endocrine disorders of reproductive-aged women. The prevalence of PCOS has been estimated at approximately 6–26%, affecting 105 million people worldwide. This systematic review aimed to synthesize the evidence on the effects of physical activity on reproductive health functions among PCOS women.

**Methods:**

The systematic review includes randomization-controlled trials (RCTs) on physical exercise and reproductive functions among women with PCOS. Studies in the English language published between January 2010 and December 2022 were identified via PubMed. A combination of medical subject headings in terms of physical activity, exercise, menstrual cycle, hyperandrogenism, reproductive hormone, hirsutism, and PCOS was used.

**Results:**

Overall, seven RCTs were included in this systematic review. The studies investigated interventions of physical activity of any intensity and volume and measured reproductive functions and hormonal and menstrual improvement. The inclusion of physical activity alone or in combination with other therapeutic interventions improved reproductive outcomes.

**Conclusion:**

The reproductive functions of women with PCOS can be improved with physical activity. Furthermore, physical activity can also reduce infertility, as well as social and psychological stress among women.

**PROSPERO systematic review registration:**

CRD42020213732.

## Introduction

Polycystic ovary syndrome (PCOS) is among the predominant endocrine disorders of reproductive-aged (18–40 years) women [1]. The prevalence of PCOS has been estimated at approximately 6–26%, which equals 105 million women affected worldwide [2]. Among reproductive-aged women, approximately 12–21% are affected by PCOS, while many remain undiagnosed [3]. The syndrome etiology is unidentified, but there is evidence of dominant X-linked inheritance involvement that is stimulated in a specific environment, including lifestyle and diet [4].

The Rotterdam criteria are widely used to diagnose PCOS with the occurrence of any two of the clinical features: oligoovulation/anovulation (O), hyperandrogenism (HA), and polycystic ovaries on ultrasound (PCOM) [5]. Based on the clinical findings and PCOS manifestations, PCOS can be categorized into four phenotypes: phenotype A (O + HA + PCOM), phenotype B (PCOM + O), phenotype C (PCOM + HA), and phenotype D (O + HA) [6].

Fertility problems are commonly manifested among PCOS women. These problems include menstrual disorders, ovulatory dysfunctions, infertility, metabolic syndrome, sarcopenic obesity, emotional distress, and physical problems [7]. The familial occurrence and symptom diversity of PCOS among different ethnicities are well established [8].

The quality of life of women with PCOS can largely be affected, as they are prone to develop psychological problems, low self-esteem, and social isolation [9]. Furthermore, women with PCOS are at risk for rapid abdominal fat deposition; approximately 60% reported being overweight or obese [10]. Reproductive and metabolic features among these PCOS women become exacerbated with obesity [11].

The recommendations in the international evidence-based guidelines for the evaluation and treatment of polycystic ovarian syndrome are based on a synthesis of the published evidence, clinical consensus, and experience. Based on how closely the true effect matches the estimated impact, the evidence is graded between extremely strong and low for PCOS assessment and management. One of the evidence-based management recommendations for PCOS is lifestyle intervention, including diet, exercise, and behavioral strategies to reduce weight, central obesity, and insulin resistance [12].

Physical activity reduces the risk of weight gain and can improve reproductive functions along with promoting quality of life among PCOS women [13]. Lifestyle modifications are key to managing PCOS. Diet and exercise are two major areas that need to be addressed for these lifestyle changes to be successful. There is a higher prevalence of polycystic ovarian morphology (PCOM) among infertile women, however, there is little evidence to indicate that PCOM alone causes serious health hazards [14]. The high prevalence of PCOS and its related complications is causing an economic burden on the healthcare system; e.g., an estimated 4.36 billion US$ was spent in the United States in 2004 to treat PCOS and its related reproductive dysfunction [15].

Despite the health benefits of physical activity/exercise in PCOS, a limited focus has been placed on analysing the independent beneficial effects of exercise on women’s reproductive functions in a systematic way. This systematic review aims to synthesize the positive effects of various exercise regimes and types on reproductive functions among PCOS women of reproductive age. Therefore, it will inform decision-makers about the importance of physical activity/exercise to address reproductive dysfunctions among PCOS women along with pharmaceutical interventions.

## Methods

The systematic review was carried out following the Preferred Reporting Items for Systematic Reviews and Meta-analysis Protocols (PRISMA) statement [16] and the Cochrane Handbook for Systematic Reviews of interventions and protocols designed for a systematic review on exercise and reproductive functions in PCOS [17]. We registered this systematic review in the PROSPERO database (November 2020, CRD42020213732) prior to data extraction. The review question was as follows: What type and intensity of exercise has effects on the reproductive functions of women with PCOS of reproductive age? The systematic review team comprised six members, including sports medicine, gynaecology, and public health.

### Study designs and participants

Randomized clinical trial (RCT) studies were selected to analyse the therapeutic interventions of physical activity/exercise in this systematic review to synthesize their effects on the reproductive functions of women with PCOS aged 13 to 45 years. English language RCT studies published in the selected databases between 1 and 2010 and 31 December 2022 were selected in this systematic review. Systematic reviews of randomized controlled trials (RCTs) are considered to be the most reliable sources of information. Despite a recent increase in the number of PCOS-controlled trials published, a systematic review to produce evidence on numerous commonly utilized treatments and approaches to PCOS management used in multiple RCT studies has not been performed.

This review article complies with the Ph.D. thesis titled “Association of physical activity and dietary habits with vitamin D and anti-Müllerian hormone among polycystic ovarian syndrome women in Lahore, Pakistan” registered at the Advanced Studies and Research Board (ref # 6067-ACAD) at the Department of Public Health, University of the Punjab, Lahore, Pakistan.

### Inclusion and exclusion criteria

RCTs use physical activity or exercise as the primary intervention to measure the relationship between cause and effect with the outcome parameters of reproductive functions (primary), menstrual irregularities, infertility, hirsutism, metabolic characteristics, and obesity (secondary). RCT studies with or without a comparison group using physical activity/exercise as the main intervention along with any other intervention were selected in this study. In the selected studies, the interventional effect should address at least one outcome parameter of reproductive functions for the post-test comparison.

Gray literature, unpublished manuscripts, conference proceedings, and publications without primary data were not selected in this systematic review. The original peer-reviewed published articles were chosen rather than the preprints. The terms “physical activity” and “fertility” were selected as the primary reproductive outcomes, and “metabolic syndrome” was selected as the secondary outcome in PCOS women. This search strategy can opt for the database with keywords searched in full text or MeSH terms, as mentioned in Table 1. Applying additional filters, such as free full text, randomized clinical trials, species humans, article language English, sex as female, and age range of 13 to 45 years, will allow the researcher to further refine your search results.

**Table 1:** Literature search using the keywords in Medical Subject Sections (MeSH)

Physical activity/exercise	Reproductive functions	PCOS
Exercise therapy Exercise training Exercise regime Physical activity Vigorous activity Moderate activity Aerobic exercise Aerobic training Resistance training	Female reproductive hormones Menstrual cycle Infertility Hyperandrogenism Hirsutism	Polycystic ovary syndrome Polycystic ovarian syndrome

Derived and modified from [17]

### Information sources and literature search

The keywords indexed in the Medical Subject Headings (MeSH) were deployed using a three-parameter search combination including exercise or physical activity (independent variable) and reproductive functions (dependent variable) and PCOS (population of interest). The research evidence was identified using PubMed. The search engines were almost accurate in finding the required studies when searching with MeSH keywords of exercise, menstrual cycle, hyperandrogenism, reproductive hormone, hirsutism, infertility, and PCOS, as shown in Table 1 [17]. A systematic search strategy was preliminarily tested to obtain the desired specificity and sensitivity.

### Study selection process

The citation manager Mendeley (desktop version 1.19.6) was used to remove duplicated studies from the identified articles. The titles and abstracts of retrieved articles were screened by one reviewer (MB) and then verified by the second reviewer (JS). The reliability of the screened and verified studies was increased by using a predefined screening form based on the eligibility criteria (level 1: screening). The full texts of the screened articles were acquired to extract information on their study design, information of participants, sample size, type of interventions and duration, follow-up period, outcome parameters, and methods used for outcome validations (level 2: selection).

**Fig. 1:**
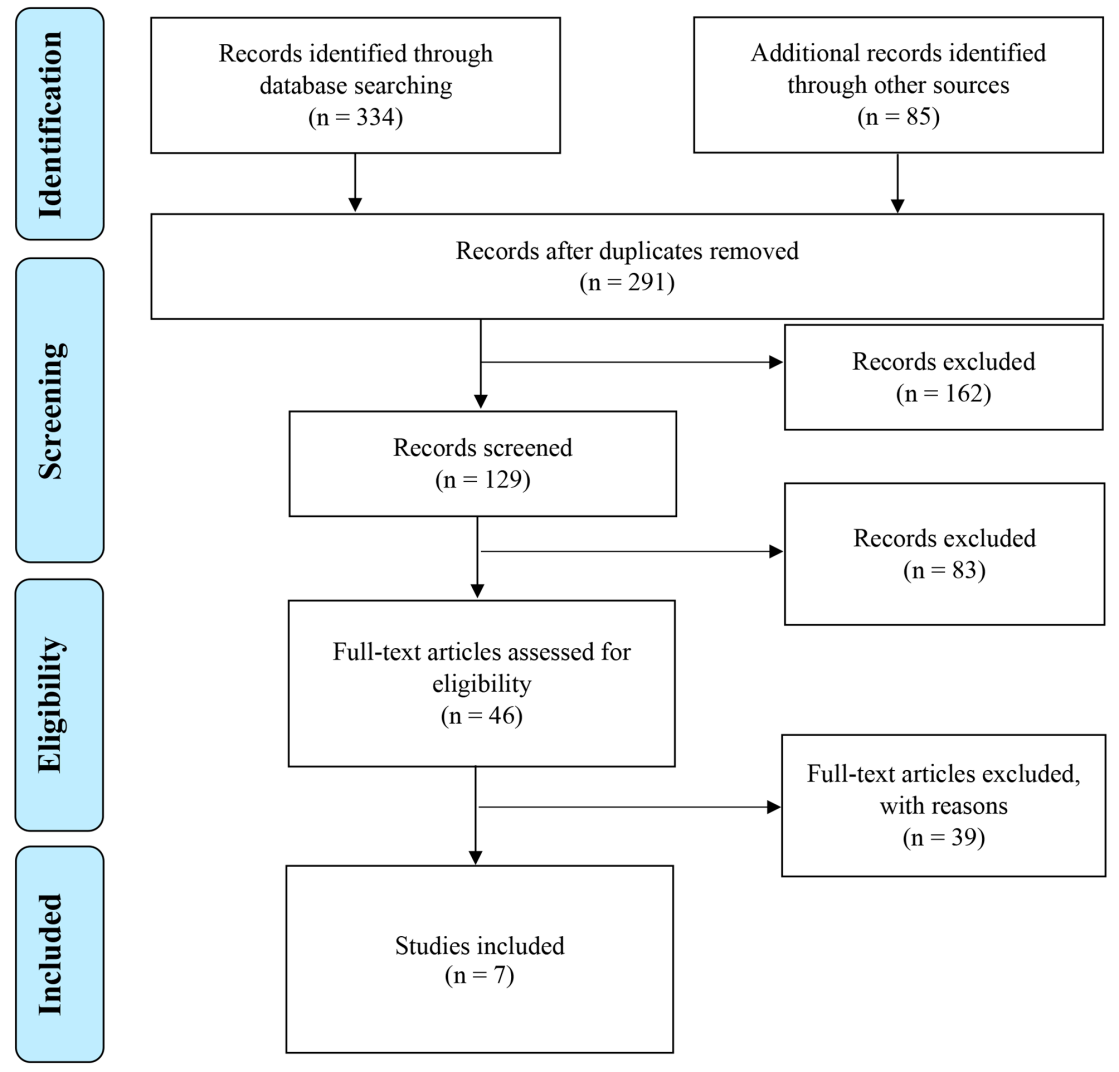
Extraction and sorting data

The appropriateness of the searched studies to be added in this systematic review was determined by identifying that the outcome measures were in the domain of interest (level 3: eligibility). All discrepancies were resolved with the mutual consensus of the reviewers (level 4: inclusion). A flow diagram related to the information on these different levels of selection was developed based on the PRISMA statements [16] and is shown in Fig. 1.

### Data items and data collection process

Two reviewers on the research team extracted the main findings from the selected articles to be included in the synthesis. Data extraction included information on study participants, intervention, comparison group, primary outcome, and research design (PICOS) to integrate all major findings. All the articles were assessed by one reviewer, while the other reviewer independently appraised 10% of randomly selected articles. All discrepancies in the findings observed by the two reviewers were resolved by mutual consensus.

General characteristics of the selected studies, including information on the author(s), year of publication, demographic characteristics, duration, and research design, were also enumerated in the PICOS tables. Study population characteristics, including the source of recruitment, sampling technique, method, age, ethnicity, and proportion of the comparison group, were considered in the data extraction process.

Reproductive functions of women with and without PCOS, including their reproductive hormone levels, metabolic characteristics, anthropometric features, and phenotype of PCOS, were extracted. The physical activity level was also considered, and data were extracted to measure the type, level, and effect of physical activity on reproductive functions.

### Risk of bias in individual studies

**Fig. 2:**
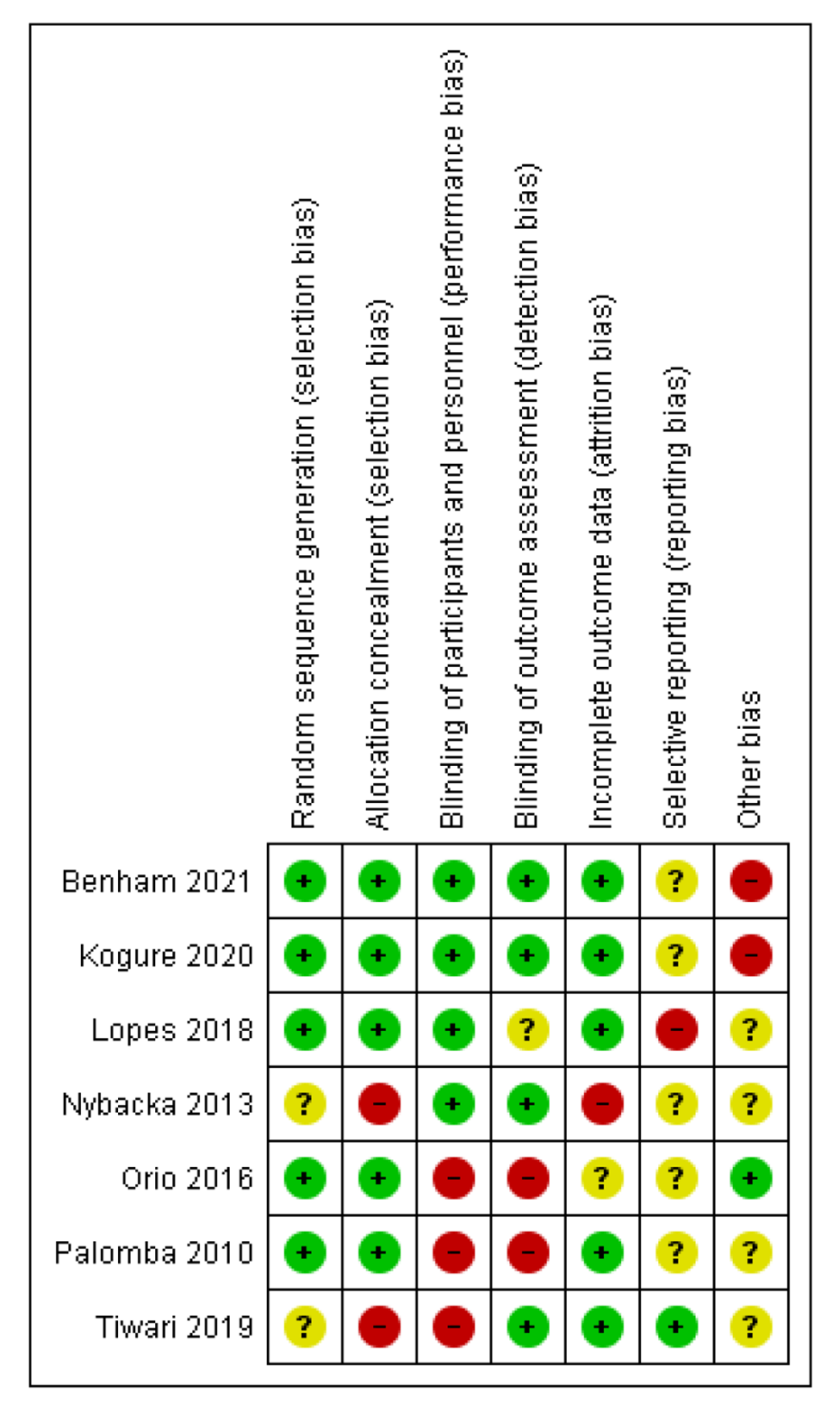
Risk of bias summary for individual studies

**Fig. 3:**
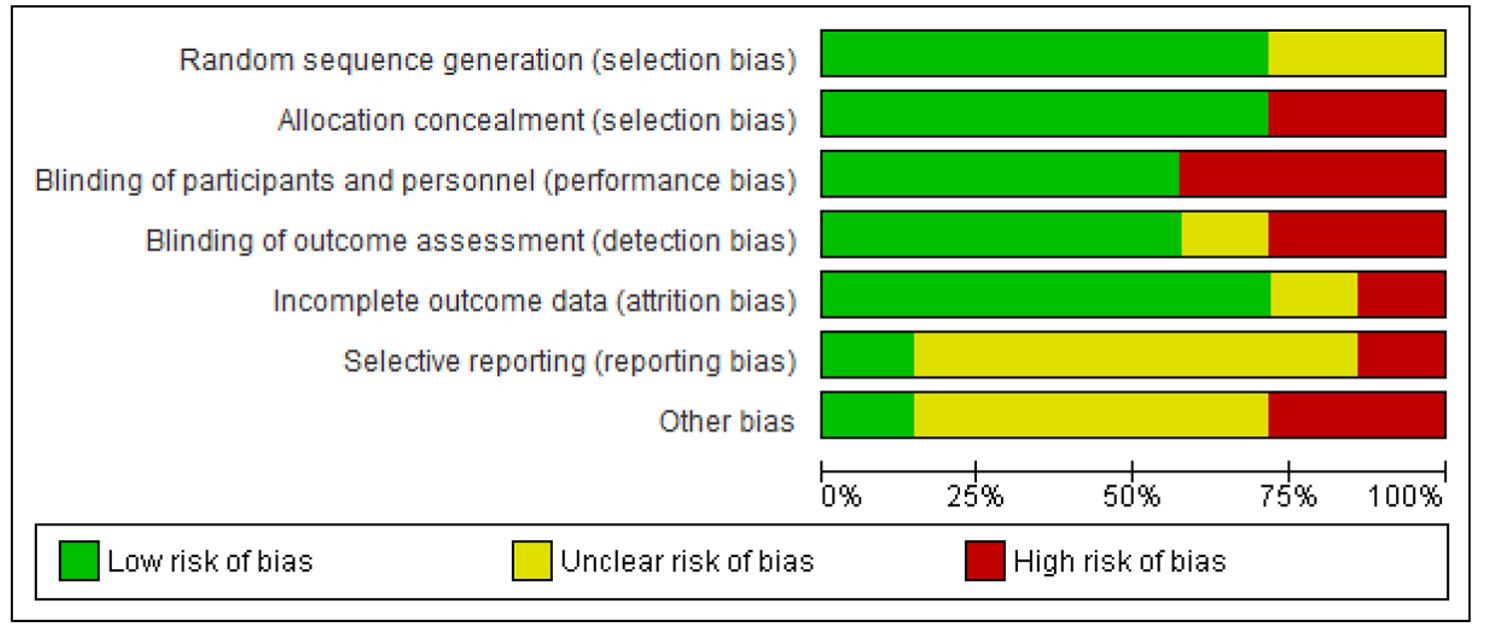
Overall risk of bias assessment

The methodological quality of the selected studies was evaluated by using the Cochrane Effective Practice and Organization of Care Risk of Bias Tool [18] and PRISMA checklist as a reference. RevMan 5.4.1 was used to generate a figure for internal validity bias risk evaluation of the included studies, as shown in Figs. 2 and 3. The databases Scopus, EMBASE, and MEDLINE were explored, but due to the restricted access, full access to the articles was not possible. In the results synthesis of this systematic review, we recognized this as a significant limitation and considered it to be a publication bias.

## Results

A total of 334 potentially eligible articles were selected for screening through PubMed. An additional 85 studies were identified through manual searches from other sources, such as Scopus. Predefined screening at level 1 excluded 128 articles, and 291 were selected after duplication was removed, as shown in Fig. 3.

At full-text analysis at level 2, 162 articles were excluded from further analysis because they did not meet the eligibility criteria based on their respective study design, information on participants, sample size, type of interventions and duration, follow-up period, outcome parameters, and methods used for outcome validations. Based on the primary and secondary outcome measures, 83 full-text articles were excluded at level 3 screening. A total of 46 articles were found eligible for full-text assessment, and 39 studies were rejected because the studies’ primary outcomes were rather concentrated on metabolic outcomes, weight loss, and quality of life among PCOS women in general and were not focused on sexual function. Among the included studies, seven RCTs were fully analysed to synthesize the evidence in this systematic review, as shown in Table 2.

**Table 2:** Benefits of physical activities on reproductive functions

Author (year)	Country	Study design	PCOS criteria	Participants (n)	Control	Age (mean ± SD) or other stated	Intervention	Reproductive health outcome measures
**Benham et al. (2021)**	Canada	Pilot randomized controlled trial	Rotterdam criteria	36	PCOS women without exercise	18–40 years	High-intensity interval training (HIIT) and Continuous aerobic exercise training (CAET),	Menstrual cycle length, and sexual function
**Kogure et al. (2020)**	Brazil	Randomized controlled trial	Rotterdam criteria	126	PCOS women without training	18–39 years	Continuous (CAT) and intermittent (IAT) aerobic training	Sexual function
**Tiwari et al. (2019)**	India	Randomized double-blinded placebo-controlled trial	Rotterdam criteria	66	PCOS women without exercise	24.46 ± 4.76 years 24.33 ± 3.89 years	Exercise for 30 min at a heart rate ≥ 120 beats/min	Menstruation frequency
**Lopes et al. (2018)**	Brazil	Randomized controlled trial	Rotterdam criteria	69	PCOS women without exercise	18–19 years	Intermittent (IAT) and continuous aerobic training (CAT)	Sexual function
**Orio et al. (2016)**	Italy	Double-blinded randomized controlled trial	Rotterdam criteria	50	PCOS women	13–44 years	Incremental cardiopulmonary exercise.	Hyperandrogenism, menstrual disturbance
**Nybacka et al. (2013)**	Sweden	Randomized controlled trial	Rotterdam criteria	57	PCOS women	18–40 years	Endurance, aerobic, and/or weight training depending on each subject’s preferences	Ovarian function, Serum Anti- Muellerian hormone
**Palomba et al. (2010)**	Italy	Randomized controlled trial	Rotterdam criteria	96	PCOS women	18–35 years	30 min on a bicycle ergometer and the exercise workload was increased gradually until a target of 60–70%	Ovulation

### Synthesis of included studies

The included studies were analysed using the PICOS model and tabulated to present the reproductive health outcomes of physical activity (Table 2). The studies are presented in descending order of their date of publication. Overall, small sample sizes were observed (range: 50–183 participants) in the included RCTs. Only one RCT [17] had a sample size ≥ 100. Most of the studies used aerobic and endurance exercises, and their related beneficial results are synthesized below.

### Exercise duration

The average duration of physical activity/exercise intervention was 15 weeks (range: 6–24 weeks). Most of the interventions were planned for 16 weeks [17–20]. Some studies planned a shorter duration of 6 weeks [19], while others had a longer duration (24 weeks) for intervention [20]. All studies reported beneficial effects of physical activity or exercise on PCOS women’s reproductive functions and menstrual frequency. The optimal exercise volume, intensity, and duration cannot be fully identified, but a minimum duration of 30 min at a submaximal heart rate level has been shown to improve reproductive functions [21].

### Exercise intensity

Most of the studies planned aerobic exercises of submaximal heart rate. Continuous and intermittent aerobic training exercise protocols as per the American College of Sports Medicine (ACSM) recommendations were followed [22]. According to the international PCOS guidelines, women of normal weight should engage in 150 min per week of moderate-intensity exercise, 75 min per week of intense exercise, or a combination of both. Obese and overweight PCOS women have been advised to engage in 250 min per week of moderate-intensity exercise, 150 min per week of intense exercise, or an equal combination of both [12].

### Effect of exercise on the perceptual body dimension

Exercise regimes including continuous aerobic training (CAT) and intermittent aerobic physical training (IAT) were designed for the experimental cohort for 16 weeks [23]. Such training programs proved to improve the satisfaction levels for their perceptual body dimension and modulate the hormone levels among PCOS women [24]. Submaximal aerobic exercises for 30 min improved BMI significantly after 3 months. The mean waist circumference was reduced by 3 cm, and the mean waist-to-hip ratio (WHR) was also significantly enhanced (0.84 ± 0.05 cm) after 6 months. The mean weight loss was approximately 1.71 ± 0.19 kg after three months and 2.5 ± 0.30 kg after six months [21]. CAT and IAT had a statistically significant reduction in the WHR after 16 weeks (p = 0.047). Similar effects were also observed among the intervention group that had cycled for 6 weeks [19].

### Effect of exercise on psychological factors

Aerobic exercise and endurance exercises also reduce the risk of anxiety and depression (P < 001) [23]. Regular physical activity has been proven to improve motivation and optimism among PCOS women [21]. Psychological factors, including anxiety and depression, were found to improve among the experimental group who took CAT and IAT for 16 weeks [25].

### Effect of exercise on sexual function

Sexual function among PCOS women improved among the experimental group taking CAT and IAT physical activity when measured on the female sexual function index (FSFI) [23]. Lopes et al. also found significant enhancement in the FSFI (p = 0.048). and its domain of satisfaction (p = 0.049) [25]. Significant improvement (72.7%) was observed in the menstrual cycle after 3 months of submaximal exercise [21]. The menstrual pattern improved among 70% of the women and shifted from amenorrhea (AM) to oligomenorrhea (OM) and normal menstruation (NM). Approximately 35% of the women also had ovulation [24]. A significant improvement in the ovulation rate was observed in the experimental group that underwent six weeks of cycling exercise intervention [19].

### Effect of exercise on hormones

Improvement in hyperandrogenism-related features was also found to be improved by CAT and IAT physical activities [23]. In another study, the testosterone level was significantly reduced among experimental groups [25]. The modified Ferriman Gallwey (mFG) score changed significantly (p < 0.003) after three months of submaximal marching exercises. Metabolic factors tend not to be improved significantly at this level of physical activity [21]. Anti-Mullerian hormone (AMH) was not significantly reduced among PCOS women who only had exercise interventions (p = 0.53) [24].

## Discussion

The beneficial effects of physical activity on PCOS women aged 13–45 years were evaluated in this systematic review. This systematic review synthesizes the effects of regular physical activity of varying volume and intensity on menstrual function and reproductive health among PCOS women. Lifestyle modification, including exercise and healthy dietary patterns, proved to be a beneficial therapy for PCOS women [26, 27]. Despite this generalized understanding of exercise effectiveness on PCOS, only a few RCTs were identified in the last decade. In the majority of studies, the control group received exercise as a secondary intervention or used exercise in combination with other interventions.

Primary and secondary outcomes of the selected RCTs vary in their endpoints. Considering these heterogeneous outcomes and their endpoints, this systematic review cannot fully synthesize the results in a homogenous manner. Selected studies mostly addressed reproductive functions [23, 25], menstrual frequency [20, 21], ovulation [19, 24], and hormonal functions [24, 25, 28].

Physical exercise can be used as an independent treatment for PCOS women to appraise all PCOS phenotypic characteristics [29]. Possible factors that are responsible for obtaining the expected response of exercise include genes, age, and hormonal status of the individual. Lifestyle changes, including physical activity modification, can be recommended as an early management strategy to reduce PCOS-related comorbidities, as it decreases insulin resistance, enhances metabolic and reproductive characteristics, and enhances self-esteem [30, 31].

Australian guidelines for the management of PCOS recommended at least 150 min of moderate-level exercise each week [10]. The time of the physical activity programs differs substantially, ranging from a few weeks to a year. PCOS is associated with overweight and obesity, but their exact prevalence among girls with PCOS is still unknown and varies according to ethnicity [32]. The different ethnic groups showed an equal benefit of physical exercise among PCOS women.

Inactivity has been associated with obesity, hypertension, peripheral insulin resistance, and dyslipidemia, as is prevalent among PCOS patients, according to the metabolic syndrome model. Women with PCOS need to take a multidisciplinary approach, including exercise [12]. Aerobic exercise can enhance glycemic control while providing a beneficial impact on sexual function and quality of life [25]. Physical activity is beneficial for PCOS women’s reproductive health because it lowers the risk of developing metabolic syndrome and its associated clinical symptoms [33].

In addition to enhancing reproductive and self-esteem in PCOS women, physical activity has positive effects on mental health [34]. It was hypothesized that exercise was an effective way to enhance the mental health of women with PCOS. The effects of changing one’s lifestyle have been proven to be astounding among PCOS women. Regular exercise enhances anthropometric measurements, reproductive biochemical outcomes, and reproductive characteristics. Numerous studies have shown that PCOS women’s health-related quality of life (HRQoL) has improved [31].

The PCOS women were found to have depression and anxiety disorders, reduced psychosexual function and self-esteem, and were prone to develop eating problems with binge eating. The suggestion indicates modifying an individual’s habits based on the screening assessment and the frequency of adverse health-related symptoms among PCOS. These interventions should focus on behavioural aspects, change dietary patterns, incorporate exercise and physical activity into daily routines, and create a management strategy to control obesity and weight [12]. A lifestyle change may prove to be both cost-effective and reduce the burden of illness.

This systematic review’s results suggested that exercise improves sexual functions and menstrual frequency and balances hormonal actions in PCOS women, especially in reducing hyperandrogenism [20] and normalizing serum anti-Mullerian hormones by optimal stimulation of ovarian follicles. Improvement in reproductive functions was observed for a maximum of 6 months and relied on the subjective responses of the participants. This could limit the effectiveness of the study outcomes, as it only considered a few ovulatory cycles. The results synthesized from a longer duration of RCTs could yield stronger results and outcomes related to exercises and reproductive functions among PCOS women.

Adolescent children are living more sedentary lifestyles due to advancements in technology. It is discovered that females in the reproductive age group do not take part in any regular physical activity. This may serve as a precursor to the development of PCOS in young women. The link between an inactive lifestyle and the development of PCOS needs to be further investigated. Future research can further investigate the role of lifestyle modification including regular physical exercise and change in dietary patterns to reduce the prevalence of PCOS and related anomalies including emotional well-being, metabolic syndrome, and self-esteem among women of the reproductive age group.

The literature regarding physical activity in patients with PCOS was found to be extremely limited. The effect size of these interventions on reproductive function outcomes was not measured, as it was beyond the scope of this systematic review. The available data were limited to fully concluding the beneficial effects of exercise on reproductive functions.

## Conclusion

The reproductive functions of women with PCOS can be improved with physical activity and can also reduce infertility and social and psychological stress among these women. The practical implication and dissemination of knowledge of strategies to improve reproductive functions and health outcomes can reduce the inappropriate costs of using health care.

## Data Availability

The datasets used and/or analysed during the current study are available from the corresponding author upon reasonable request.

## Abbreviations

ACSM: American College of Sports Medicine
AM: Amenorrhea
AMH: Anti-Mullerian Hormone
CAT: Continuous Aerobic Training
EPOC: Effective Practice and Organization of Care
FSFI: Female Sexual Function Index
HRQoL: Health-Related Quality of Life
IAT: Intermittent Aerobic Physical Training
MeSH: Medical Subject Headings
NM: Normal menstruation
OM: Oligomenorrhea
PCOS: Polycystic ovarian syndrome
WHR: Waist-hip ratio
